# Investigating the presence of surgical learning in the Timing of Primary Surgery for cleft palate randomised trial

**DOI:** 10.1177/17407745241302488

**Published:** 2025-01-10

**Authors:** Elizabeth J Conroy, Jane M Blazeby, Girvan Burnside, Jonathan A Cook, Carrol Gamble

**Affiliations:** 1 Liverpool Clinical Trials Centre, University of Liverpool, Liverpool, UK; 2 Oxford Clinical Trials Research Unit, Centre for Statistics in Medicine, Nuffield Department of Orthopaedics, Rheumatology and Musculoskeletal Sciences, University of Oxford, Oxford, UK; 3 Centre for Surgical Research, Bristol NIHR Biomedical Research Centre, University Hospitals Bristol and Weston NHS Foundation Trust and Population Health Sciences, University of Bristol, Bristol, UK; 4 Surgical Interventions Trials Unit, Nuffield Department of Orthopaedics, Rheumatology and Musculoskeletal Sciences, University of Oxford, Oxford, UK

**Keywords:** Randomised controlled trial, surgical learning, surgery, cleft palate, statistics, statistical modelling, change over time, fistula, operation time

## Abstract

**Background/aims:**

When conducting a randomised controlled trial in surgery, it is important to consider surgical learning, where surgeons’ familiarity with one, or both, of the interventions increases during the trial. If present, learning may compromise trial validity. We demonstrate a statistical investigation into surgical learning within a trial of cleft palate repair.

**Methods:**

The Timing of Primary Surgery compared primary surgery, using the Sommerlad technique, for cleft palate repair delivered at 6 or 12 months of age. Participating surgeons had varying levels of experience with the intervention and in repair across the age groups. Trial design aimed to reduce the surgical learning via pre-trial surgical technique training and balancing the randomisation process by surgeon. We explore residual learning effects by applying visual methods and statistical models to a surgical outcome (fistula formation) and a process indicator (operation time).

**Results:**

Notably, 26 surgeons operated on 521 infants. As the trial progressed, operation time reduced for surgeons with no pre-trial Sommerlad experience (n = 2), before plateauing at 30 operations, whereas it remained stable for those with prior experience. Fistula rates remained stable regardless of technique experience. Pre-trial age for primary surgery experience had no impact on either measures.

**Conclusion:**

Managing learning effects through design was not fully achieved but balanced between trial arms, and residual effects were minimal. This investigation explores the presence of learning, within a randomised controlled trial that may be valuable for future trials. We recommend such investigations are undertaken to aid trial interpretation and generalisability, and determine success of trial design measures.

## Introduction

When designing and analysing a randomised controlled trial (RCT) in surgery, it is important to consider potential differences in expertise between participating surgeons. Methods may aim to achieve balance at trial outset but surgical expertise may change during the trial, as participating surgeons become more familiar with one, or both, of the trial interventions. This learning can happen within or outside of the trial.

The presence of learning can compromise the validity of the trial if the expertise of the surgeon is skewed towards the better established, more widely used or easy to perform technique.^1–3^ Learning can continue over a long time for some techniques, perhaps hundreds of procedures, and attaining the experience required prior to running a trial may not be possible in some specialties.^4,5^ Design features, such as including surgeon in the randomisation strategy, defining prerequisite levels of surgical experience or providing pre-trial training, can minimise any learning impact but may not eradicate it entirely.^6^ A statistical description of any learning curve effect is one way to explore learning within a trial.^6,7^

TOPS, Timing Of Primary Surgery for cleft palate, was a parallel, international two-arm, multicentre RCT comparing primary surgery, using the Sommerlad technique,^8^ for isolated cleft palate repair at 6 or 12 months of age. Notably, 558 infants were recruited from 23 centres in Brazil, Denmark, Norway, Sweden and the United Kingdom. Independent ethics committees in each country approved the protocol and amendments. Infants were randomised (1:1) to undergo surgery at age 6 or 12 months. The trial design has been reported previously.^9,10^

TOPS concluded that those in the 6 months group were less likely to have velopharyngeal insufficiency at age 5 years than the 12 months group (risk ratio, 0.59; 95% confidence interval: 0.36–0.99; p = 0.04). Complications were infrequent and similar in both groups, and while the need for secondary surgery was similar between groups, the reasons varied. The results, in detail, are reported elsewhere.^9^

Learning was an important consideration during trial design. The trial used minimisation to balance the randomised groups within surgeon. In addition, all surgeons participated in extensive pre-trial training to standardise surgical delivery.^10^

However, despite careful consideration at the design stage not all aspects of palatal repair are amendable to standardisation.^11–13^ Furthermore, international recruitment amplified aspects further due to differing geographies, introducing differences in healthcare systems and surgical training. Despite steps to design out any learning effect, we aim to investigate any residual learning within the study and consider impact on trial conclusions.

## Methods

### Considering learning within TOPS

**Figure 1.**
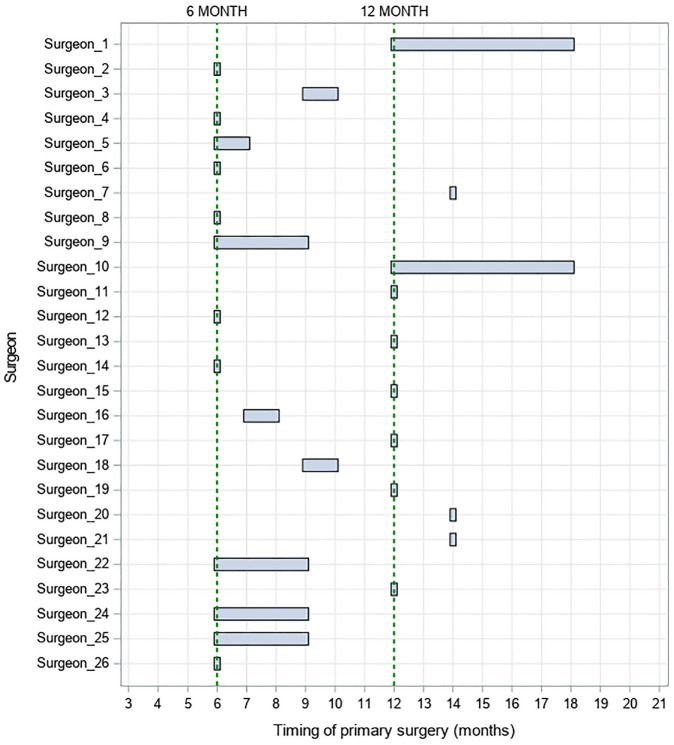
TOPS for cleft palate at the time of grant application per surgeon.

The opening of the mouth is smaller in younger infants, making it more difficult to conduct the operation. Younger infants, at the time of operating, may therefore be more prone to scarring or complications. Prior to TOPs, timing of surgery was known to vary across recruiting centres, from 6 to 18 months of age. No surgeon had experience in both intervention timings, see Figure 1 and Table 1. Surgeons within centres who routinely operate at age 6 months may find surgery at 12 months more difficult, or vice versa. It may also be argued that surgeons who routinely operated on infants at 9 months may find alternative timings challenging in both directions. Balance, within operating surgeon, was achieved through incorporating operating surgeon as a stratification factor in the randomisation process. Of the total 23, 2 recruiting centres shared surgical infrastructure and operated within a single shared unit. Of the 22 surgical centres, 19 had one consultant surgeon, two surgical centres had two and the remaining centre had three, see Table 1.

**Table 1.** Summary of prior experience of centres and surgeons.

Centre	Prior experience (months)	Technique experience	Surgeon	Sample size	Primary centre	Secondary centre
Centre 1	12–18	No	Surgeon 1	69	69	0
			Surgeon 10	85	85	0
Centre 2	14	Yes	Surgeon 7	19	18	1
			Surgeon 20	8	7	1
			Surgeon 21	17	17	0
Centre 3	6	Yes	Surgeon 4	41	40	1
Centre 4	9–10	Yes	Surgeon 3	37	37	0
Centre 5	12	Yes	Surgeon 13	20	20	0
			Surgeon 15	5	5	0
Centre 6	12	Yes	Surgeon 17	25	25	0
Centre 7	6–9	Yes	Surgeon 9	21	21	0
Centre 8	9–10	Yes	Surgeon 18	20	20	0
Centre 9	6	Yes	Surgeon 12	22	22	0
Centre 10	6–7	Yes	Surgeon 5	22	22	0
Centre 11	12	Yes	Surgeon 19	22	19	3
Centre 12	7–8	Yes	Surgeon 16	14	14	0
Centre 13	12	Yes	Surgeon 23	12	12	0
Centre 14	6	Yes	Surgeon 2	11	11	0
Centre 15	6	Yes	Surgeon 8	11	11	0
Centre 16	6–9	Yes	Surgeon 25	9	8	1
Centre 17	6-9	Yes	Surgeon 24	5	5	0
Centre 18	12	Yes	Surgeon 11	6	6	0
Centre 19	6	Yes	Surgeon 14	6	6	0
Centre 20	6–9	Yes	Surgeon 22	7	4	3
Centre 21	6	Yes	Surgeon 6	4	4	0
Centre 22	6	Yes	Surgeon 26	3	3	0

In addition to concerns around expertise relating to age at surgery, one centre had no experience of the Sommerlad technique prior to participation, see Table 1.

### Mitigating the impact of learning through design

There are several theories suggesting that surgeons with higher case volumes achieve better outcomes. In addition, individual skill and the complexity of protocols are significant factors contributing to surgical success.^12,14^ In TOPS, defining a minimum number of operations as a prerequisite for participation, at levels defined in other fields of surgery, was not possible. This is because the amount of experience required, using other specialties as a basis for estimating, was unrealistic for many surgeons within participating countries.^5^ Therefore, prior to participation, all participating surgeons undertook a formal programme of surgical standardisation of the technique. The programme included direct technique instruction, within the operating theatre, from the surgeon who developed the technique, written instruction and video demonstrations on how to perform the procedure, an illustrated seminar and a discussion session. Surgeons were officially endorsed as competent by the technique’s developer prior to trial participation.^10^

### Defining measures of learning and experience

Three variables were defined at the surgeon level to represent preexisting, or changing, experience. The first, *operation sequence* (or *X1*), is a count variable indicating the order infants had been treated within trial surgeon that represents increasing experience. Counting trial operations is the closest proxy to a time-varying learning effect when no information on non-randomised cases is available.^1,7^ The second, *technique experience* (or *X2*), is a binary variable indicating whether the surgeon had experience in delivering the Sommerlad technique prior to trial participation. The third, *age experience* (or *X3*), is a continuous variable indicating routine age of the child (in months) at surgery, or median age where a range is given, at the centre in which the surgeon is based.

Two measures of surgical learning, previously used within the cleft literature, were selected.^5,12^ The first, a continuous variable, is *operation time* (or *Y1*) and is a measure of surgical learning used across conditions.^1,7,15^ The second, as a patient outcome of surgical repair is *occurrence of fistula* (or *Y2*). It is a dichotomous outcome and defined as in the original trial.^16^

### Statistical principles and visual methods

A complete case analysis approach is used, adopting the intention-to-treat principle.^17^ No imputation methods for missing data were applied. However, 95% confidence intervals are presented throughout and p-values, applied to learning variables, were nominally assessed at 5% significance level. All analyses were undertaken using the SAS software (version 9.4) with SAS/STAT package 14.3. (Statistical Analysis Software (SAS^®^) 9.1.4; SAS Institute Inc., Cary, NC, USA).

The number of infants randomised, undergoing surgery and followed for outcome data are presented. The hierarchical nature of the data, the number of infants within surgeon and within centre are summarised.

Continuous data are presented as means, standard deviations and overall range to be consistent with available methodology to explore learning over time. In the presence of skewed data, a log transformation is used. *Operation time* is presented within surgeon and split by treatment arm. Between-surgeon variation is explored using box and funnel plots. For the latter, a target operation time of the overall surgeon mean observed is assigned together with two prediction limits, at 95% and 99.8%, to identify outlying areas of differing extremity. Prediction limits are calculated using the appropriate z-scores for the line represented and each possible *N.*^18^ Within-surgeon variation is explored using moving average plots, of Order 5, for surgeons performing at least 20 primary operations.^1^

Binary data are presented as frequencies and percentages. *Occurrence of fistula* is presented within surgeon and split by treatment arm. Between-surgeon variation is explored using the funnel plots.^18^ For surgeons performing at least 20 trial operations, within-surgeon variation is investigated using the cumulative sum (cusum) plots, a graphical method for identifying trends in data.^1^ In the plots, the x-axis represents the *operation sequence* and the y-axis is a performance indicator based on the within-surgeon data series. Predetermined proficiency levels, representing the desirable performance levels in cleft literature, of 85% and 90%, are assumed.^1,19,20^ When interpreting the plots, surgeons operating at the defined proficiency have a flat cusum line, a decreasing line, if exceeding the proficiency level and increasing line, if operating at a lower proficiency.^1^

### Statistical modelling

*Operation time* (*Y1*) is analysed using a two-level multilevel linear models and *occurrence of fistula* (*Y2*) using a two-level multilevel logistic model. Operating surgeon was included as a random effect and treatment (6 and 12 months) as a fixed effect in all models.

Three models were applied to each outcome. The first, *Model A* (unadjusted), contains a treatment covariate only, representing an analysis approach ignoring any potential learning effect, the approach used in the original trial analysis.^16^ The second, *Model B* (adjusted for learning and technique), contains a treatment covariate and experience variables *operation sequence* (*X1*) and *technique experience* (*X2*). This model adjusts for experience gained throughout the trial and whether the surgeon had experience with the Sommerlad technique prior to participation. The third, *Model C* (adjusted for learning and routine infant age), contains a treatment covariate and experience variables *operation sequence* (*X1*) and *age experience* (*X3*). This model adjusts for experience gained throughout the trial and the age of the infant that the surgeon routinely operated on prior to participation.

The differences between models are that *Model A* is unadjusted for learning, *Model B* adjusts for learning and surgeon baseline experience of the surgical technique, while *Model C* adjusts for learning and surgeon baseline experience of age of infant at primary operation.

The introduction of interaction terms, between experience variables, is considered based on exploratory analysis identified through visual methods. *Supplementary Box 1* provides more details.

## Results

### Summary of dataset

Notably, 26 surgeons delivered the TOPS operations. Six operated across two centres, with the number of operations in the secondary centre being few compared to that of the primary (two surgeons with three operations in secondary centre, four surgeons with one). However, 11 surgeons operated on at least 20 participants (median cluster size: 15.5). Table 1 and Supplementary Table 1 provide further details.

Notably, 521 of the 558 randomised infants (93.4%) had surgery (6 months: 266; 12 months: 255). *Occurrence of fistula* was available for all infants. *Operation time* was available for 516 patients, see Table 2 for further details.

**Table 2.** Summary of TOPS outcomes.

		6 months	12 months	Overall
No. randomised	N	281 (50.4)	277 (49.6%)	558
No. with surgery data	n (%)	266 (51.1%)	255 (48.9%)	521 (93.4%)
Operation time	n (%)	264 (51.2%)	252 (48.9%)	516 (92.5%)
	M (SD)	86.3 (38.2)	85.9 (35.7)	84.7 (37.0)
	[Min, Max]	[30.0, 245.0]	[30.0, 210.0]	[30.0, 245.0]
	Missing	2 (0.8%)	3 (1.2%)	5 (1.0%)
Fistula				
Yes	n (%)	40 (15.0%)	33 (12.9%)	73 (14.0%)
No	n (%)	186 (85.0%)	222 (87.1%)	448 (86.0%)

### Operation time: a measure of surgical process

Operation time, split by surgeon and timing of surgery, is summarised in Supplementary Table 2.

Between-surgeon variability is presented visually in Supplementary Figure 1. The plot demonstrates substantial variation in the average operation time between surgeons. The plot does not indicate a decrease in variability with increasing number of operations. Operation time, split by treatment group, for each surgeon according to their pre-participation timing experience is presented in Supplementary Figure 2. This plot indicates little difference at the surgeon level in the mean operation times between treatment arms.

For within-surgeon differences, Surgeons 1 and 10 had no experience with the Sommerlad technique prior to participating in the trial although each had delivered a greater number of palate repairs than other participating surgeons. The moving average plots for these surgeons suggested a downwards trend in operation time, with these surgeons getting faster at performing the technique during the trial by approximately 1 hr, see Supplementary Figure 3. This trend was evident for both treatment arms, despite both surgeons operating on infants aged 12 to 18 months prior to participation, see Figure 1.

Surgeons experienced in the Sommerlad technique did not show any clear trend over time, overall or by treatment group, see Supplementary Figure 3. Intra-surgeon operation times largely remained stable yet some inter-surgeon differences in average time, and variability, were observed. Operation times did not appear to change within surgeon during the trial.

Table 3 presents the modelling results, see Supplementary Box 1 more detail on the models applied. *Model A* (unadjusted) shows that operating on infants aged 6 months takes approximately 7.1 min longer than at 12 months (β = 7.104; 95% CI: 3.047–11.161; p = 0.0006).

**Table 3.** Operation time (Y1): results of model fitting.

Outcome	Variables	Coefficient	95% CI	p
Operation time (Y1)	Model A			
	Treatment: 6 months surgery	7.104	3.047 to 11.161	0.0006
	Model B			
	Treatment: 6 months surgery	4.435	0.548 to −8.321	0.0254
	Operation sequence (X1)	−0.568	−0.709 to −0.427	<0.0001
	Technique experience: Yes (X2)	−31.113	−67.934 to 5.709	0.0975
	Model B with interaction term			
	Treatment: 6 months surgery	5.310	1.506 to 9.115	0.0063
	Operation sequence (X1)	−0.727	−0.878 to −0.576	<0.0001
	Technique experience: Yes (X2)	−43.442	−81.802 to −5.082	0.0265
	X1 × X2^a^	0.885	0.537 to 1.233	<0.001
	Model C			
	Treatment: 6 months surgery	4.476	0.590 to 8.362	0.0240
	Operation sequence (X1)	−0.561	−0.700 to −0.421	<0.0001
	Age experience (X3)	2.435	−0.677 to 5.548	0.1249

Reference level for treatment: 12 months surgery; Reference level for technical experience: No experience; CI: Confidence Interval.

a Interaction term of *Operation sequence* (*X1*) and *Technique experience: Yes* (*X2*) to indicate if operation times during the trial are different for surgeons with and without prior technique experience.

*Model B* (adjusted for learning and technique), which extends *Model A* to incorporate *operation sequence* and *technique experience*, indicates that operation time decreases by approximately 1 min for every two operations performed (β = 0.568; 95% CI: −0.709 to −0.427; p < 0.0001). When an interaction term between learning variables is included, all variables in the model (*treatment, sequence, technique experience* and interaction) are significant at the 0.05 level. Indicating that the slopes of the regression lines between *sequence* and *operation time* are different for surgeons with and without prior *technique experience*. However, the confidence intervals around the coefficient, *technique experience*, are wide and this is reflective of the underlying distribution of operation times demonstrating wide variation between surgeons, particularly Surgeons 1 and 10.

*Model C* (adjusted for learning and routine infant age), which extends *Model A* to incorporate *operation sequence* and *age experience*, indicates that, like *Model B*, operation time decreases by approximately 1 min for every two trial operations performed (β = −0.561; 95% CI: −0.700 to −0.421; p < 0.0001) but *age experience* does not alter operation time (β = 2.435; 95% CI: −0.677 to 5.548; p = 0.1249).

### Fistula: an outcome of surgical success

Fistula rate, split by surgeon and timing of surgery, is summarised for the 521 infants with complete outcome data in Supplementary Table 3.

Between-surgeon variability is presented visually in Supplementary Figure 4. Surgeon 14 is the only surgeon with a fistula rate who exceeds the upper boundary of the 95% confidence interval; however, the number of operations within this surgeon was low (n = 6) in comparison to other trial surgeons. There is clear variability of surgeon fistula rates which is not explained by the number of operations.

For within-surgeon differences, Surgeon 1, who had no prior Sommerlad experience, the cusum plot indicates an initial period to approximately 15 operations where performance is below the proficiency rate. The curve then plateaus with proficiency maintained for the remainder of the trial. Surgeon 10 shows variability with an initial high period of proficiency for approximately 35 operations, followed by a period of steep deterioration for approximately 15 operations regaining proficiency for the remaining trial operations. The plots split by treatment group indicate that the period of poorer outcomes tends to be within the 12 months surgery group, despite these surgeons having prior experience in delivering surgery at this age prior to participation. There were no clear trends within the cusum plots. Surgeons 5, 9, 13 and 17, each with differing background experience, showed little change in fistula rate during the trial. Cusum plots for all surgeons are presented in Supplementary Figure 5.

Table 4 presents the modelling results. Supplementary Box 1 provides more detail on the models applied. *Model A* (unadjusted) shows that there was no significant difference between the fistula rates within the two treatment groups, although the confidence intervals are wide (OR: 1.187; 95% CI: 0.720 to 1.957; p = 0.5008).

**Table 4.** Occurrence of fistula (Y2): results of model fitting.

Variables	Coefficient	95% CI	p
Model A			
Treatment: 6 months surgery	1.187	0.720 to 1.957	0.5008
Model B			
Treatment: 6 months surgery	1.135	0.683 to 1.886	0.6245
Operation sequence (X1)	0.991	0.441 to 2.822	0.3567
Technique experience: Yes (X2)	1.115	0.441 to 2.822	0.8177
Model C			
Treatment: 6 months surgery	1.134	0.683 to 1.882	0.6269
Operation sequence (X1)	0.991	0.974 to 1.008	0.2914
Age experience (X3)	0.984	0.898 to 1.882	0.7249

Reference level for treatment: 12 months surgery; Reference level for technical experience: No experience; CI: Confidence Interval.

*Model B* (adjusted for learning and technique), again incorporating *operation sequence* and *technique experience*, indicates that neither covariate is a predictive factor of fistula (*Operation sequence:* OR: 0.991; 95% CI: 0.441 to 2.822; p = 0.3567; *Technique experience:* OR: 1.115; 95% CI: 0.441 to 2.822; p = 0.8177). There does not appear to be a change in fistula rate over time during the trial as indicated visually by the cusum plots, see Supplementary Figure 4 and so further exploration of interaction terms was not warranted.

*Model C* (adjusted for learning and routine infant age), again incorporating *operation sequence* and *age experience*, indicates neither term is statistically significant (*Operation sequence* (*X1*): OR: 0.991; 95% CI: 0.974 to 1.008; p = 0.2914; *Age experience* (*X3*): OR: 0.984; 95% CI: 0.898 to 1.882; p = 0.7249). These results are consistent with that modelling on operation time.

## Discussion

This analysis of learning within a trial showcases methods for evaluating the presence of learning across two different measures, using the TOPS trial as a practical example of a surgical RCT. The potential for learning was raised at trial design and, to reduce variability in delivery, a pre-trial training programme was completed by all participating surgeons to support surgical standardisation. This investigation demonstrates that, despite extensive training in the technique, a level of surgical learning remained (demonstrated by decreasing operation time), but importantly that the variation observed did not extend to trial outcomes (fistula).

Due to pre-trial variations in surgeon experience, within-trial learning could come from two sources: technique experience, in using the Sommerlad technique, and age experience, defined as routine care infant age at surgery. Presence of learning, and any impact, was explored for two outcomes recognised as measures of cleft surgical learning: operation time, an outcome of surgical process, and occurrence of fistula, a patient outcome of surgical success.^5,12^

There was evidence of a learning curve for operation time, a process outcome. Surgeons with pre-trial technique experience operated faster and demonstrated little change in delivery during the trial. Surgeons with no technique experience started slower but got quicker during the trial, and the rate varied by treatment arm. The surgeons with no pre-trial experience of using the technique, Surgeon 1 and Surgeon 10, both had prior expertise in operating on infants aged 12–18 months. They achieved a plateau within both treatment timings by approximately 20 operations but had very different average operating times. Importantly, these changes did not appear to affect the fistula rate, a patient outcome and trial secondary outcome. Provided there is no compromise on safety, and a stable safety profile is demonstrated by our analysis of fistula rate here and the main trial findings,^9^ a shorter operation time is also beneficial as it is less expensive, less time required by the surgeon and the rest of the operative team.^20^ It is estimated that a reduction of 3 min in average turnover times would result in a 0.8% reduction in staffing costs.^21^ There are also safety benefits associated with reduced time under a general anaesthetic, such as reduced risk of intraoperative blood loss and infection exposure.^22^

Importantly, age experience, the pre-trial age that the surgeon delivered primary surgery, did not affect operation time or occurrence of fistula. Aside from Surgeons 1 and 10, the exploratory moving average and cusum plots showed no differences between surgeons regardless of age experience, and this finding was further supported using statistical models. This finding is crucial as indicated that, in a trial where age at surgery is the intervention, any pre-trial differences in expertise did not appear to have impacted either outcome explored.

This reanalysis of the TOPS data has a number of limitations. First, available data were limited to surgical procedures conducted as part of the TOPS trial and as such experience gained during but outside trial conduct is not captured. This is common across trials and, unless the procedure is limited to the trial or the infrastructure is in place support the collection of cases outside of the trial, the assessment is complicated by incomplete case series for the surgeons participating. Second, the number of procedures undertaken by operating surgeons prior to participation was not collected. This would have provided a stronger basis on which to measure experience. Third, infants were randomised before they are 6 months of age to surgery at 6 or 12 months. Therefore, for each surgeon, the initial number of surgeries would have been on infants aged 6 months with final operations being on infants at 12 months. Therefore, by trial design, 6 months learning curve may have plateaued before any observed in the 12 months arm. Fourth, when interpreting the impact of technique experience on outcomes, the surgeons with no prior experience were from the same unit. Therefore, differences in outcomes observed may be due to different protocols rather than differences due to surgical skill. Two surgeons in particular had very similar fistula rates, despite their overall operating time differing by approximately 1 hr. Fifth, the variable used to indicate routine age of the child (in months) at surgery was defined at the centre level in which the surgeon is based as a proxy for surgeon expertise. Finally, identification of learning is reduced in surgeons who contributed fewer operations to the study. However, the 11 surgeons who with a reasonable sample size, showed relatively steady outcomes over time and those contributing the most operations, Surgeons 1 and 10 who each operated on 69 and 85 operations, respectively, demonstrated a learning curve for the process outcome.

The limitations identified are typical of surgical trials. The strengths of this article are that it illustrates how relatively simple techniques can be applied to investigate learning curve effects using data collected routinely in trials of this type. The need for transparency around learning curves is highlighted within the guidelines on reporting nonpharmacologic interventions.^23^ The trends observed in operation time (as a measure of surgical process) were not mirrored in occurrence of fistula (as a measure of surgical success), raising the question of whether there is a need for better measures of learning, such as surgeon reported satisfaction with the intervention or infection. The outcomes chosen and presented here represent those identified within the literature as indicators of surgery success both within, and beyond, the cleft field.^7,12,14^ Furthermore, they are routinely collected, making them a pragmatic choice in trials where learning is not the primary trial objective, but may have the potential to confound results if not explored.

Surgeon experience has long been attributed as a cause of variation in cleft surgery outcome, but until this investigation, there has been no analysis of the surgical learning curve within a controlled environment to support this.^5,11,12^ Considerations for surgical learning in key trial publications are often unclear within the surgical literature.^15^ The TOPS trial, despite aiming to diminish learning effects through standardisation of the technique and the randomisation process, showed some evidence that there was a change in operating time during the trial as surgeons became more experienced with the trial technique. With experience, surgery took less time and short-term outcomes were unaffected. This method could be used in future trials as an aid to explore whether learning is occurring in the trial and be valuable when interpreting trial results. Methods are applicable to other complex healthcare interventions where healthcare professional skills are important in the intervention delivery (e.g. psychological therapies) or where there is a systematic effect of time, such as seasonal or circadian variation.^24^

## Supplemental Material

sj-docx-1-ctj-10.1177_17407745241302488 – Supplemental material for Investigating the presence of surgical learning in the Timing of Primary Surgery for cleft palate randomised trial Supplemental material, sj-docx-1-ctj-10.1177_17407745241302488 for Investigating the presence of surgical learning in the Timing of Primary Surgery for cleft palate randomised trial by Elizabeth J Conroy, Jane M Blazeby, Girvan Burnside, Jonathan A Cook and Carrol Gamble in Clinical Trials
