# Supplementary material for: Investigating the presence of surgical learning in the Timing of Primary Surgery for cleft palate randomised trial
Source: Clin Trials. 2025 Jan 10;22(2):200–8. doi: 10.1177/17407745241302488 (PMC11986075; doi:10.1177/17407745241302488)
Supplement: sj-docx-1-ctj-10.1177_17407745241302488 – Supplemental material for Investigating the presence of surgical learning in the Timing of Primary Surgery for cleft palate randomised trial [file sj-docx-1-ctj-10.1177_17407745241302488.docx]

**SUPPLEMENTAL DIGITAL CONTENT**

[Supplementary Box 1: Statistical modelling 2](#_Toc158131516)

Supplementary Table 1: Summary of surgeon clusters 6

[Supplementary Table 2: Summary of operation time by surgeon in TOPS 7](#_Toc158131518)

[Supplementary Figure 1: Funnel plot of average operation time by number of operations 11](#_Toc158131506)

[Supplementary Figure 2: Box plot of operation time by treatment timing by surgeon 12](#_Toc158131507)

[Supplementary Figure 3: Moving average operation time against operation sequence 13](#_Toc158131508)

[Supplementary Table 3: Summary of fistula by surgeon in TOPS 22](#_Toc158131519)

[Supplementary Figure 4: Funnel plot of fistula by number of operations 26](#_Toc158131509)

[Supplementary Figure 5: Cusum charts for occurrence of fistula against operation sequence 27](#_Toc158131510)

Supplementary Box 1: Statistical modelling

Statistical models were applied to explore the impact of experience variables: *operation sequence (X1), technique experience (X2),* and *age experience (X3)* on outcome. Operating surgeon was included as a random effect and treatment (six-months, twelve-months) as a fixed effect in all models.

*Operation time (Y1)* was analysed using a two-level multilevel linear model, reflective of the two-level data structure of patient (level 1) within surgeon (level 2).

$y_{ij}=\beta_{0}+\beta_{1}x_{ij}+\ldots+u_{j}+\epsilon_{ij}$ where $u_{j}\sim N(0,\sigma_{u}^{2})$ and $\epsilon_{ij}\sim N(0,\sigma_{\epsilon}^{2})$

Equation 1

Where $y_{ij}$ indicates the outcome for the $i$-th patient, $\beta_{0}$ is an intercept, $\beta_{1}$represents treatment and $\ldots$ represents additional terms added to specific models applied, specifically: s*equence (X1)* as a patient level covariate ($\beta_{2}x_{ij}$) and *technique experience (X2)* and *age experience (X3)* as surgeon level covariates ($\beta_{3}x_{j}$ and $\beta_{4}x_{j}$ respectively).

*Occurrence of fistula (Y2)* was analysed using a two-level multilevel logistic model, reflective of the two-level data structure of patients (level 1) within surgeon (level 2).

$\log\left( \frac{\pi_{ij}}{1-\pi_{ij}} \right)=\beta_{0}+\beta_{1}x_{ij}+\ldots+u_{j}$ where $u_{j}\sim N(0,\sigma_{u}^{2})$

Equation 2

Where $\pi_{ij}$ indicates the probability of the $i$-th patient having the event and other covariates are as defined in *Equation 1*.

Three models were considered for each outcome. *Model A,* which contained a treatment covariate only, represents an analysis approach ignoring any potential learning effect. *Model B* included treatment and experience variables *operation sequence (X1)* and *technique experience* *(X2),* which adjusts for any potential trend due to experience gained throughout the trial and whether the surgeon had experience with the technique prior to participation. *Model C* included treatment and experience variables *operation sequence (X1)* and *age experience* *(X3),* which adjusts for any potential trend due to experience gained throughout the trial and the age of the infant that the surgeon routinely operated on prior to participation. The introduction of interaction terms, between experience variables, were considered based on exploratory analysis indicating further trends.

Supplementary Table 1: Summary of surgeon clusters

|  | TOPS |
| --- | --- |
| Sample size | 552 |
| No. with surgery data | 521 (94.4%) |
| No. of surgeon clusters | 26 |
| Median cluster size | 15.5 |
| No. at least 20 patients | 11 (42.3%) |

Supplementary Table 2: Summary of operation time by surgeon in TOPS

Totals presented based only on infants with operation time data (N=516, see *Table 1*).

|  |  | Operation time | | |
| --- | --- | --- | --- | --- |
|  |  | Overall | Six-months | Twelve-months |
| Overall | Surgeries | 516 | 264 | 252 |
|  | Mean (SD) | 84.7 (37.7) | 86.3 (38.2) | 82.9 (35.7) |
|  | [Min, Max] | [30.0, 245.0] | [30.0, 245.0] | [30.0, 210.0] |
| Surgeon 1 | Surgeries | 69 | 30 | 39 |
|  | Mean (SD) | 133.6 (33.9) | 143.5 (36.2) | 125.9 (30.0) |
|  | [Min, Max] | [80, 245] | [82, 245] | [80, 210] |
| Surgeon 2 | Surgeries | 11 | 5 | 6 |
|  | Mean (SD) | 147.5 (37.2) | 148.0 (44.4) | 147.0 (34.5) |
|  | [Min, Max] | [109, 213] | [109, 213] | [112, 200] |
| Surgeon 3 | Surgeries | 37 | 19 | 18 |
|  | Mean (SD) | 68.7 (11.1) | 70.3 (11.0) | 67.1 (11.3) |
|  | [Min, Max] | [50, 90] | [52, 90] | [50, 90] |
| Surgeon 4 | Surgeries | 41 | 22 | 19 |
|  | Mean (SD) | 52.2 (9.6) | 52.9 (9.3) | 51.4 (10.2) |
|  | [Min, Max] | [37, 75] | [37, 70] | [38, 75] |
| Surgeon 5 | Surgeries | 22 | 12 | 10 |
|  | Mean (SD) | 49.8 (11.6) | 53.3 (13.0) | 45.6 (8.4) |
|  | [Min, Max] | [30, 75] | [36, 75] | [30, 55] |
| Surgeon 6 | Surgeries | 4 | 1 | 3 |
|  | Mean (SD) | 70.5 (17.5) | 53 (.) | 76.3 (16.0) |
|  | [Min, Max] | [53, 92] | [53, 53] | [60, 92] |
| Surgeon 7 | Surgeries | 18 | 9 | 9 |
|  | Mean (SD) | 74.3 (12.4) | 74.1 (13.4) | 74.6 (12.1) |
|  | [Min, Max] | [56, 99] | [57, 99] | [56, 91] |
| Surgeon 8 | Surgeries | 11 | 6 | 5 |
|  | Mean (SD) | 91.6 (16.4) | 98 (17.5) | 84.0 (12.4) |
|  | [Min, Max] | [65, 125] | [75, 125] | [65, 95] |
| Surgeon 9 | Surgeries | 21 | 13 | 8 |
|  | Mean (SD) | 60.3 (15.3) | 65.8 (13.9) | 51.3 (13.6) |
|  | [Min, Max] | [30, 90] | [50, 90.0] | [30, 65] |
| Surgeon 10 | Surgeries | 85 | 44 | 41 |
|  | Mean (SD) | 63.3 (29.1) | 69.4 (33.8) | 56.7 (21.5) |
|  | [Min, Max] | [32, 165] | [32, 165] | [34, 137] |
| Surgeon 11 | Surgeries | 6 | 2 | 4 |
|  | Mean (SD) | 102.8 (23.2) | 107.0 (24.0) | 100.8 (24.2) |
|  | [Min, Max] | [80, 139] | [90, 124] | [80, 139] |
| Surgeon 12 | Surgeries | 22 | 11 | 11 |
|  | Mean (SD) | 103.8 (24.6) | 103.2 (17.4) | 104.4 (31.1) |
|  | [Min, Max] | [65, 165] | [75, 120] | [65, 165] |
| Surgeon 13 | Surgeries | 20 | 12 | 8 |
|  | Mean (SD) | 64.2 (12.8) | 65.8 (10.0) | 61.8 (16.7) |
|  | [Min, Max] | [49, 101] | [54, 83] | [49, 101] |
| Surgeon 14 | Surgeries | 6 | 3 | 3 |
|  | Mean (SD) | 93.3 (18.4) | 93.3 (6.5) | 93.3 (28.4) |
|  | [Min, Max] | [68, 124] | [87, 100] | [68, 124] |
| Surgeon 15 | Surgeries | 5 | 1 | 4 |
|  | Mean (SD) | 111.4 (23.0) | 147.0 (.) | 102.5 (13.3) |
|  | [Min, Max] | [90, 147] | [147, 147] | [90, 121] |
| Surgeon 16 | Surgeries | 14 | 6 | 8 |
|  | Mean (SD) | 87.7 (24.0) | 86.7 (33.5) | 88.5 (16.2) |
|  | [Min, Max] | [43, 135] | [43, 135] | [65, 105] |
| Surgeon 17 | Surgeries | 25 | 12 | 13 |
|  | Mean (SD) | 66.1 (23.0) | 69.6 (24.1) | 62.8 (22.4) |
|  | [Min, Max] | [30, 129] | [43, 129] | [30, 112] |
| Surgeon 18 | Surgeries | 19 | 10 | 9 |
|  | Mean (SD) | 98.8 (24.2) | 104.3 (26.8) | 92.8 (20.7) |
|  | [Min, Max] | [60, 165] | [84, 165] | [60, 135] |
| Surgeon 19 | Surgeries | 22 | 12 | 10 |
|  | Mean (SD) | 125.8 (24.7) | 128.3 (26.1) | 122.7 (24.0) |
|  | [Min, Max] | [95, 180] | [95, 180] | [98, 180] |
| Surgeon 20 | Surgeries | 8 | 5 | 3 |
|  | Mean (SD) | 99.0 (37.4) | 90.6 (43.5) | 113 (25.5) |
|  | [Min, Max] | [52, 165] | [52, 165] | [88, 139] |
| Surgeon 21 | Surgeries | 15 | 8 | 7 |
|  | Mean (SD) | 108.3 (17.1) | 109.4 (16.0) | 107.0 (19.5) |
|  | [Min, Max] | [85, 149] | [85, 136] | [93, 149] |
| Surgeon 22 | Surgeries | 6 | 3 | 3 |
|  | Mean (SD) | 51.5 (18.0) | 44.7 (12.9) | 58.3 (22.5) |
|  | [Min, Max] | [30, 80] | [30, 54] | [35, 80] |
| Surgeon 23 | Surgeries | 12 | 6 | 6 |
|  | Mean (SD) | 81.5 (11.8) | 86.0 (13.9) | 77.0 (8.2) |
|  | [Min, Max] | [60, 100] | [60, 100] | [67, 89] |
| Surgeon 24 | Surgeries | 5 | 4 | 1 |
|  | Mean (SD) | 59.0 (9.8) | 59.0 (11.3) | 59.0 (.) |
|  | [Min, Max] | [45, 70] | [45, 70] | [59, 59] |
| Surgeon 25 | Surgeries | 9 | 6 | 3 |
|  | Mean (SD) | 78.9 (16.8) | 81.7 (20.0) | 73.3 (7.6) |
|  | [Min, Max] | [63, 105] | [63, 105] | [65, 80] |
| Surgeon 26 | Surgeries | 3 | 2 | 1 |
|  | Mean (SD) | 91.3 (4.0) | 91.0 (5.7) | 92 (.) |
|  | [Min, Max] | [87, 95] | [87, 95] | [92, 92] |

Supplementary Figure 1: Funnel plot of average operation time by number of operations

The target is the overall surgeon mean operation time of 84.7 minutes. The 95% and 99.8% prediction limits around the overall mean operation time are presented. The within surgeon observed mean operation time is plotted against the number of TOPS operations for that surgeon.


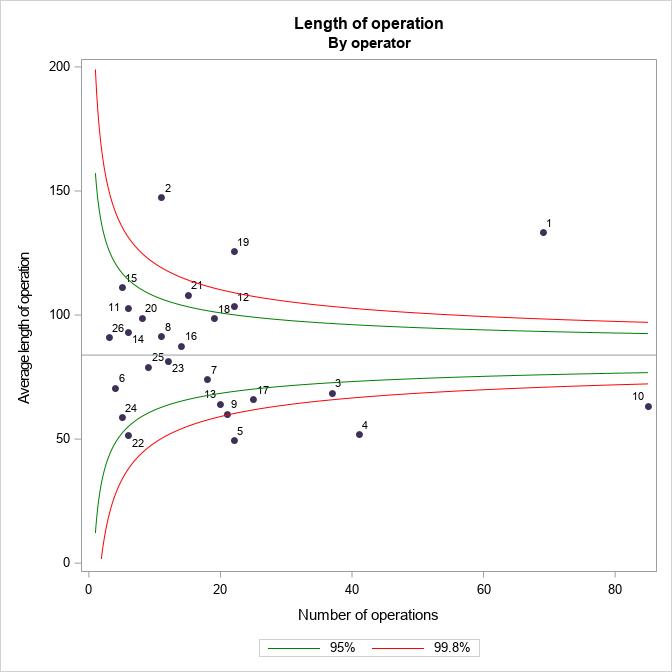


Supplementary Figure 2: Box plot of operation time by treatment timing by surgeon

The circle represents the mean. Within the box, the midline represents the median and the bottom and top edges the inter-quartile range. The whiskers represent the minimum and maximum values for surgeon. The x-axis is ordered by increasing timing expertise (l-r).


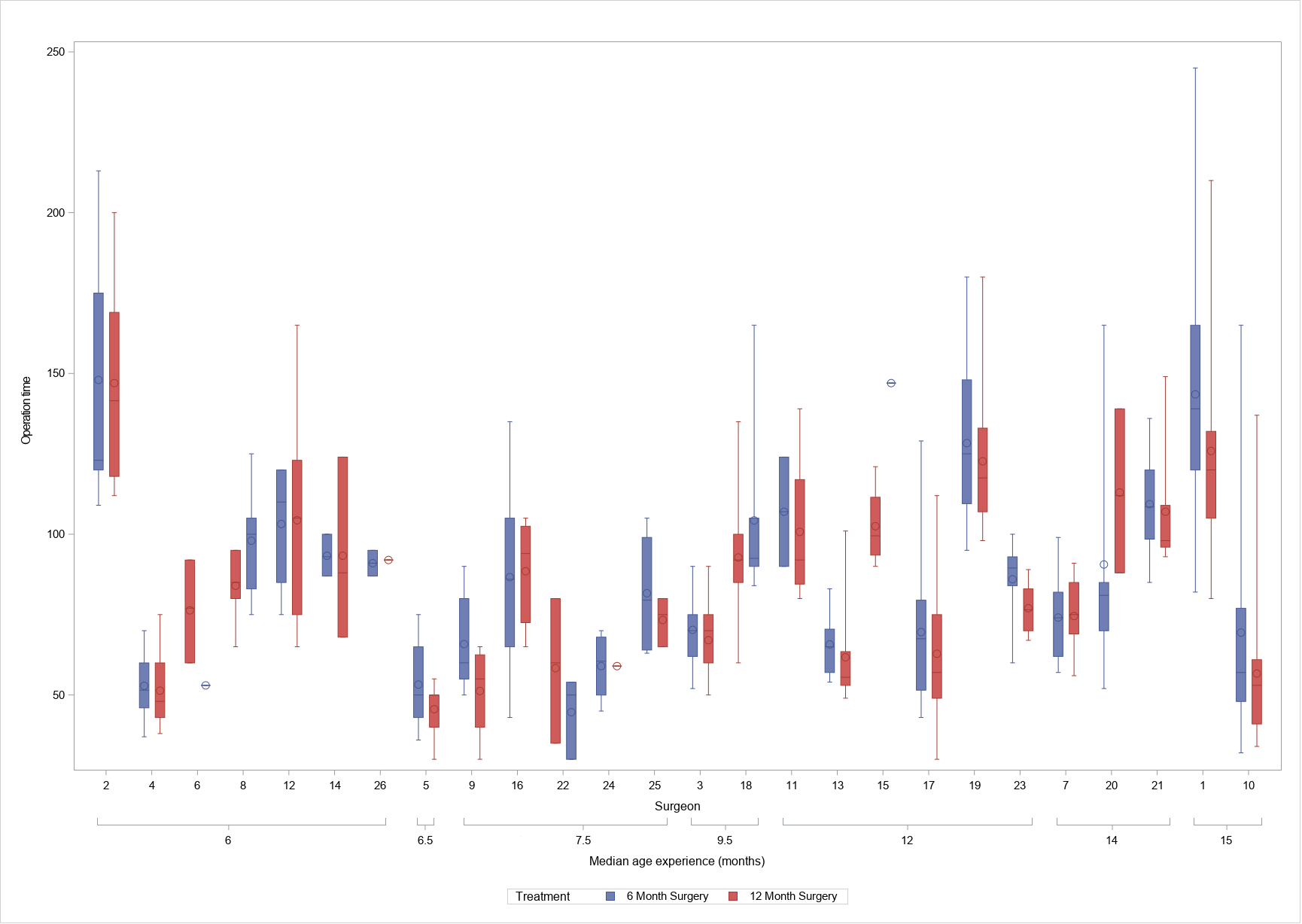


Supplementary Figure 3: Moving average operation time against operation sequence

Figures presented for surgeons who operated on at least twenty patients only. Moving averages of order five, based on an equal weight distribution, presented.

*Supplementary Figure 3 (continued): Moving average operation time against operation sequence*

*Supplementary Figure 3 (continued): Moving average operation time against operation sequence*

Figures presented for surgeons who operated on at least twenty patients only. Moving averages of order five, based on an equal weight distribution, presented.

*Supplementary Figure 3 (continued): Moving average operation time against operation sequence*

Figures presented for surgeons who operated on at least twenty patients only. Moving averages of order five, based on an equal weight distribution, presented.

*Supplementary Figure 3 (continued): Moving average operation time against operation sequence*

Figures presented for surgeons who operated on at least twenty patients only. Moving averages of order five, based on an equal weight distribution, presented.

*Supplementary Figure 3 (continued): Moving average operation time against operation sequence*

Figures presented for surgeons who operated on at least twenty patients only. Moving averages of order five, based on an equal weight distribution, presented.

*Supplementary Figure 3 (continued): Moving average operation time against operation sequence*

Figures presented for surgeons who operated on at least twenty patients only. Moving averages of order five, based on an equal weight distribution, presented.

*Supplementary Figure 3 (continued): Moving average operation time against operation sequence*

Figures presented for surgeons who operated on at least twenty patients only. Moving averages of order five, based on an equal weight distribution, presented.

*Supplementary Figure 3 (continued): Moving average operation time against operation sequence*

Figures presented for surgeons who operated on at least twenty patients only. Moving averages of order five, based on an equal weight distribution, presented.

*Supplementary Figure 3 (continued): Moving average operation time against operation sequence*

Figures presented for surgeons who operated on at least twenty patients only. Moving averages of order five, based on an equal weight distribution, presented.

*Supplementary Figure 3 (continued): Moving average operation time against operation sequence*

Figures presented for surgeons who operated on at least twenty patients only. Moving averages of order five, based on an equal weight distribution, presented.

Supplementary Table 4: Summary of fistula by surgeon in TOPS

Totals presented based only on infants with surgery data (N=521).

|  |  | **Fistula** | | |
| --- | --- | --- | --- | --- |
|  |  | Overall | Six-months | Twelve-months |
| Overall | Surgeries | 521 | 266 | 255 |
|  | Fistula (%) | 73 (14.0%) | 40 (15.0%) | 33 (12.9%) |
|  | [95% CI] | [11.0, 17.0] | [10.7, 19.3] | [8.8, 17.1] |
| Surgeon 1 | Surgeries | 69 | 30 | 39 |
|  | Fistula (%) | 9 (13.0%) | 4 (13.3%) | 5 (12.8%) |
|  | [95% CI] | [5.1, 21.0] | [1.2, 25.5] | [2.3, 23.3] |
| Surgeon 2 | Surgeries | 11 | 5 | 6 |
|  | Fistula (%) | 0 (0.0%) | 0 (0.0%) | 0 (0.0%) |
|  | [95% CI] | [., .] | [., .] | [., .] |
| Surgeon 3 | Surgeries | 37 | 19 | 18 |
|  | Fistula (%) | 7 (18.9%) | 4 (21.1%) | 3 (16.7%) |
|  | [95% CI] | [6.3, 31.5] | [2.7, 39.4] | [0.0, 33.9] |
| Surgeon 4 | Surgeries | 41 | 22 | 19 |
|  | Fistula (%) | 8 (19.5%) | 5 (22.7%) | 3 (15.8%) |
|  | [95% CI] | [7.4. 31.6] | [5.2, 40.2] | [0.0, 32.2] |
| Surgeon 5 | Surgeries | 22 | 12 | 10 |
|  | Fistula (%) | 2 (9.1%) | 2 (16.7%) | 0 (0.0%) |
|  | [95% CI] | [0.0, 21.1] | [0.0, 37.8] | [., .] |
| Surgeon 6 | Surgeries | 4 | 1 | 3 |
|  | Fistula (%) | 0 (0.0%) | 0 (0.0%) | 0 (0.0%) |
|  | [95% CI] | [., .] | [., .] | [., .] |
|  |  |  |  |  |
|  |  |  |  |  |
| Surgeon 7 | Surgeries | 19 | 10 | 9 |
|  | Fistula (%) | 0 (0.0%) | 0 (0.0%) | 0 (0.0%) |
|  | [95% CI] | [., .] | [., .] | [., .] |
| Surgeon 8 | Surgeries | 11 | 6 | 5 |
|  | Fistula (%) | 3 (27.3%) | 3 (50.0%) | 0 (0.0%) |
|  | [95% CI] | [1.0, 53.6] | [10.0, 90.0] | [., .] |
| Surgeon 9 | Surgeries | 21 | 13 | 8 |
|  | Fistula (%) | 2 (9.5%) | 2 (15.4%) | 0 (0.0%) |
|  | [95% CI] | [0.0, 22.1] | [0.0, 35.0] | [., .] |
| Surgeon 10 | Surgeries | 85 | 44 | 41 |
|  | Fistula (%) | 8 (9.4%) | 4 (9.1%) | 4 (9.8%) |
|  | [95% CI] | [3.2, 15.6] | [0.6, 17.6] | [0.7, 18.8] |
| Surgeon 11 | Surgeries | 6 | 2 | 4 |
|  | Fistula (%) | 0 (0.0%) | 0 (0.0%) | 0 (0.0%) |
|  | [95% CI] | [., .] | [., .] | [., .] |
| Surgeon 12 | Surgeries | 22 | 11 | 11 |
|  | Fistula (%) | 4 (18.2%) | 1 (9.1%) | 3 (27.3%) |
|  | [95% CI] | [2.1, 34.3] | [0.0, 26.1] | [1.0, 53.6] |
| Surgeon 13 | Surgeries | 20 | 12 | 8 |
|  | Fistula (%) | 2 (10.0%) | 1 (8.3%) | 1 (12.5%) |
|  | [95% CI] | [0.0, 23.1] | [0.0, 24.0] | [0.0, 35.4] |
| Surgeon 14 | Surgeries | 6 | 3 | 3 |
|  | Fistula (%) | 3 (50.0%) | 1 (33.3%) | 2 (66.7%) |
|  | [95% CI] | [10.0, 90.0] | [0.0, 86.7] | [13.3, 100.0] |
| Surgeon 15 | Surgeries | 5 | 1 | 4 |
|  | Fistula (%) | 1 (20.0%) | 0 (0.0%) | 1 (25.0%) |
|  | [95% CI] | [0.0, 55.1] | [., .] | [0.0, 67.4] |
| Surgeon 16 | Surgeries | 14 | 6 | 8 |
|  | Fistula (%) | 3 (21.4%) | 2 (33.3%) | 1 (12.5%) |
|  | [95% CI] | [0.0, 42.9] | [0.0, 71.1] | [0.0, 35.4] |
| Surgeon 17 | Surgeries | 25 | 12 | 13 |
|  | Fistula (%) | 2 (8.0%) | 1 (8.3%) | 1 (7.7%) |
|  | [95% CI] | [0.0, 18.6] | [0.0, 24.0] | [0.0, 22.2] |
| Surgeon 18 | Surgeries | 20 | 10 | 10 |
|  | Fistula (%) | 0 (0.0%) | 0 (0.0%) | 0 (0.0%) |
|  | [95% CI] | [., .] | [., .] | [., .] |
| Surgeon 19 | Surgeries | 22 | 12 | 10 |
|  | Fistula (%) | 6 (27.3%) | 2 (16.7%) | 4 (40.0%) |
|  | [95% CI] | [8.7, 45.9] | [0.0, 37.8] | [9.6, 70.4] |
| Surgeon 20 | Surgeries | 8 | 5 | 3 |
|  | Fistula (%) | 3 (37.5%) | 2 (40.0%) | 1 (33.3%) |
|  | [95% CI] | [4.0, 71.0] | [0.0, 82.9] | [0.0, 86.7] |
| Surgeon 21 | Surgeries | 17 | 9 | 8 |
|  | Fistula (%) | 5 (29.4%) | 2 (22.2%) | 3 (37.5%) |
|  | [95% CI] | [7.8, 51.1] | [0.0, 49.4] | [4.0, 71.0] |
| Surgeon 22 | Surgeries | 7 | 3 | 4 |
|  | Fistula (%) | 2 (28.6%) | 1 (33.3%) | 1 (25.0%) |
|  | [95% CI] | [0.0, 62.0] | [0.0, 86.7] | [0.0, 67.4] |
| Surgeon 23 | Surgeries | 12 | 6 | 6 |
|  | Fistula (%) | 1 (8.3%) | 1 (16.7%) | 0 (0.0%) |
|  | [95% CI] | [0.0, 24.0] | [0.0, 46.5] | [., .] |
| Surgeon 24 | Surgeries | 5 | 4 | 1 |
|  | Fistula (%) | 1 (20.0%) | 1 (25.0%) | 0 (0.0%) |
|  | [95% CI] | [0.0, 55.1] | [0.0, 67.4] | [., .] |
| Surgeon 25 | Surgeries | 9 | 6 | 3 |
|  | Fistula (%) | 1 (11.1%) | 1 (16.7%) | 0 (0.0%) |
|  | [95% CI] | [0.0, 31.6] | [0.0, 46.5] | [., .] |
| Surgeon 26 | Surgeries | 3 | 2 | 1 |
|  | Fistula (%) | 0 (0.0%) | 0 (0.0%) | 0 (0.0%) |
|  | [95% CI] | [., .] | [., .] | [., .] |

Supplementary Figure 4: Funnel plot of fistula by number of operations

The target was the overall surgeon fistula rate of 14.0%. The 95% and 99.8% prediction limits around the overall fistula rate are presented. The within surgeon observed fistula rate is plotted against the number of TOPS operations for that surgeon.


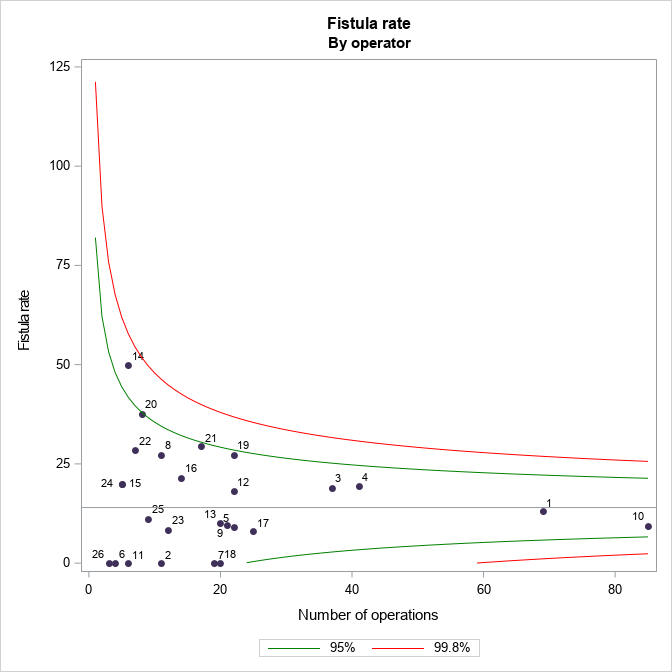


Supplementary Figure 5: Cusum charts for occurrence of fistula against operation sequence

Figures presented for surgeons who operated on at least twenty patients only.

*Supplementary Figure 4 (continued): Cusum charts for occurrence of fistula against operation sequence*

Figures presented for surgeons who operated on at least twenty patients only.

*Supplementary Figure 4 (continued): Cusum charts for occurrence of fistula against operation sequence*

Figures presented for surgeons who operated on at least twenty patients only.

*Supplementary Figure 4 (continued): Cusum charts for occurrence of fistula against operation sequence*

Figures presented for surgeons who operated on at least twenty patients only.

*Supplementary Figure 4 (continued): Cusum charts for occurrence of fistula against operation sequence*

Figures presented for surgeons who operated on at least twenty patients only.

*Supplementary Figure 4 (continued): Cusum charts for occurrence of fistula against operation sequence*

Figures presented for surgeons who operated on at least twenty patients only.

*Supplementary Figure 4 (continued): Cusum charts for occurrence of fistula against operation sequence*

Figures presented for surgeons who operated on at least twenty patients only.

*Supplementary Figure 4 (continued): Cusum charts for occurrence of fistula against operation sequence*

Figures presented for surgeons who operated on at least twenty patients only.

*Supplementary Figure 4 (continued): Cusum charts for occurrence of fistula against operation sequence*

Figures presented for surgeons who operated on at least twenty patients only.

*Supplementary Figure 4 (continued): Cusum charts for occurrence of fistula against operation sequence*

Figures presented for surgeons who operated on at least twenty patients only.

*Supplementary Figure 4 (continued): Cusum charts for occurrence of fistula against operation sequence*

Figures presented for surgeons who operated on at least twenty patients only.
